# Usefulness of MRI targeted prostate biopsy for detecting clinically significant prostate cancer in men with low prostate-specific antigen levels

**DOI:** 10.1038/s41598-021-00548-4

**Published:** 2021-11-09

**Authors:** Seokhwan Bang, Jiwoong Yu, Jae Hoon Chung, Wan Song, Minyong Kang, Hyun Hwan Sung, Hwang Gyun Jeon, Byong Chang Jeong, Seong Il Seo, Hyun Moo Lee, Seong Soo Jeon

**Affiliations:** Department of Urology, Samsung Medical Center, Sungkyunkwan University School of Medicine, 81 Irwon-ro, Gangnam-gu, Seoul, 06351 Republic of Korea

**Keywords:** Cancer, Urology

## Abstract

We aimed to evaluate the detection rates of prostate cancer (PCa) and clinically significant PCa (csPCa) using magnetic resonance imaging-targeted biopsy (MRI-TBx) in men with low prostate-specific antigen (PSA) levels (2.5–4.0 ng/mL). Clinicopathologic data of 5502 men with PSA levels of 2.5–10.0 ng/mL who underwent transrectal ultrasound-guided biopsy (TRUS-Bx) or MRI-TBx were reviewed. Participants were divided into four groups: LP-T [low PSA (2.5–4.0 ng/mL) and TRUS-Bx, *n* = 2018], LP-M (low PSA and MRI-TBx, *n* = 186), HP-T [high PSA (4.0–10.0 ng/mL) and TRUS-Bx, *n* = 2953], and HP-M (high PSA and MRI-TBx, *n* = 345). The detection rates of PCa and csPCa between groups were compared, and association of biopsy modality with detection of PCa and csPCa in men with low PSA levels were analyzed. The detection rates of PCa (20.0% vs. 38.2%; *P* < 0.001) and csPCa (11.5% vs. 32.3%; *P* < 0.001) were higher in the LP-M group than in the LP-T group. Conversely, there were no significant differences in the detection rates of PCa (38.2% vs. 43.2%; *P* = 0.263) and csPCa (32.3% vs. 39.4%; *P* = 0.103) between the LP-M and HP-M groups. Multivariate analyses revealed that using MRI-TBx could predict the detection of csPCa (odds ratio 2.872; 95% confidence interval 1.996‒4.132; *P* < 0.001) in men with low PSA levels. In summary, performing MRI-TBx in men with low PSA levels significantly improved the detection rates of PCa and csPCa as much as that in men with high PSA levels.

## Introduction

The introduction of prostate-specific antigen (PSA) testing has facilitated early detection of prostate cancer (PCa) in recent decades, leading to a downward migration in the stage of newly diagnosed PCa^1^. In addition, there have been several reports that approximately half of the PCa diagnosed in men with PSA levels lower than 4.0 ng/mL had clinically aggressive characteristics, suggesting that the conventional PSA cutoff value should be lowered^2,3^. However, there are also concerns that the lower PSA cutoff value could result in overdiagnosis and overtreatment of PCa. In Western populations, PCa overdiagnosis in screen-detected cases has been reported with estimated values ranging from 22 to 42%^4,5^.

In Germany, Paschen, U et al. reported there was no benefit nor harm in PSA screening^6^. Collectively, these challenges have led to a need for more selective modalities in the diagnosis of PCa.

Since the mid-2010s, modalities such as prebiopsy multiparametric magnetic resonance imaging (mpMRI) have emerged as adjunctive diagnostic strategies to improve the detection of clinically significant PCa (csPCa)^7–10^. In particular, three important studies (i.e., PRECISION^11^, 4M^12^, and MRI-FIRST^13^) of prebiopsy mpMRI and MRI-targeted biopsy (MRI-TBx) have indicated that MRI-TBx is 2–12% more accurate for detecting csPCa than transrectal ultrasound-guided biopsy (TRUS-Bx), and could reduce 11%–14% of the overdiagnosis of indolent PCa. However, as these studies were based on populations with a median PSA level of 6–7 ng/mL, the impact of MRI-TBx in men with much lower PSA levels was not fully investigated.

Previous work from our group showed that the detection rate of PCa using TRUS-Bx and the pathologic characteristics of prostatectomy specimens were comparable between men with low PSA (2.5–4.0 ng/mL) and high PSA (4.0–10.0 ng/mL) levels^14^. This led to our institution adopting a PSA cutoff value of 2.5 ng/mL for prostate biopsy in 2008. After setting the cutoff value, MRI-TBx was added as a new biopsy modality. Therefore, we conducted a consecutive study on the clinical performance of MRI-TBx in men with low PSA levels. In this study, we aimed to compare MRI-TBx with TRUS-Bx in men with low PSA levels (2.5–4.0 ng/mL) in terms of the detection rates of PCa and csPCa. Simultaneously, we compared the detection rates of PCa and csPCa using each biopsy modality in men with low PSA with those in men with high PSA levels (4.0–10.0 ng/mL).

## Results

**Figure 1:**
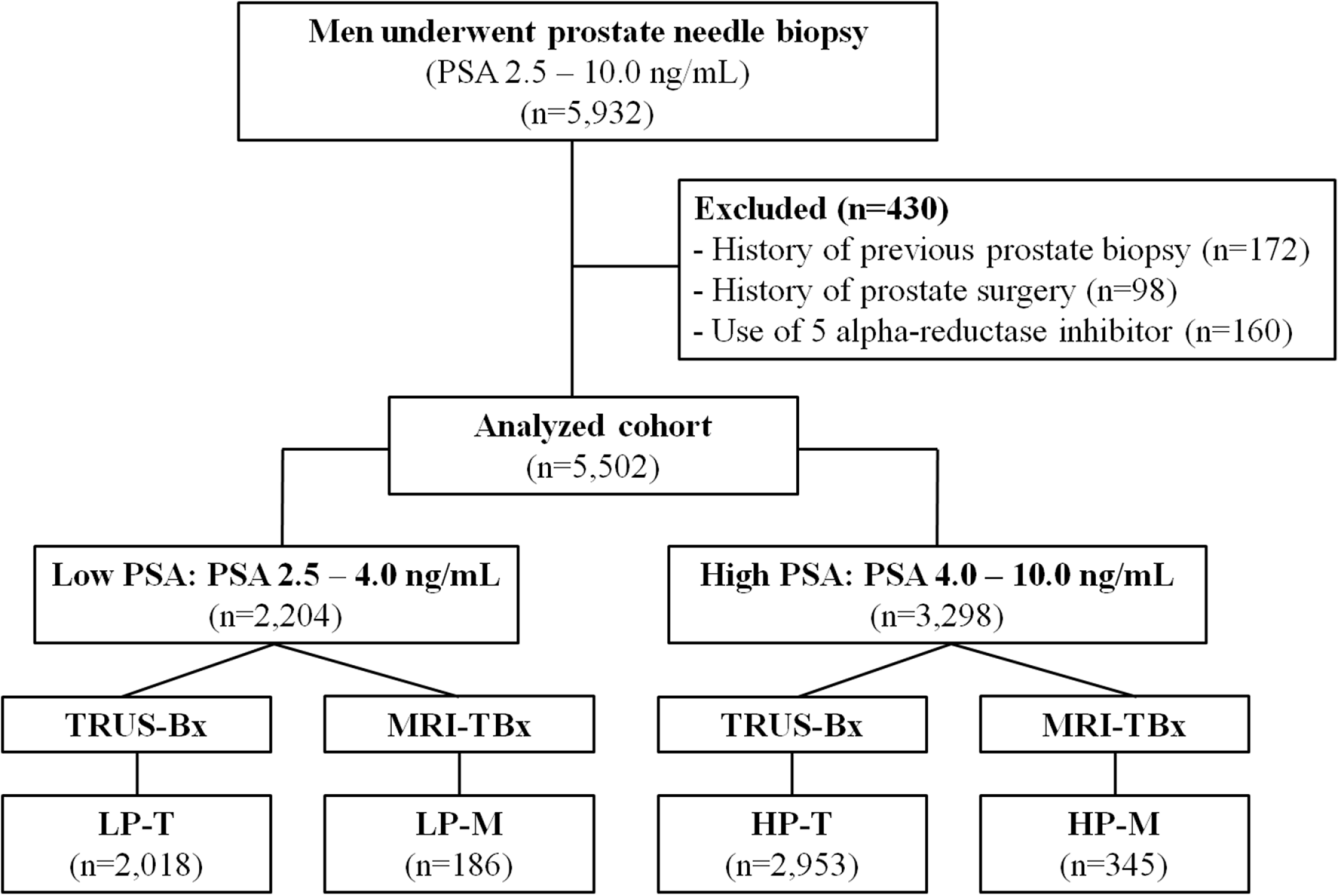
Flowchart of patients who met the study inclusion criteria. *TRUS-Bx* Transrectal ultrasound-guided biopsy, *MRI-TBx* Magnetic resonance imaging-targeted biopsy. Groups: LP-T (men with PSA 2.5–4.0 ng/mL who underwent TRUS-Bx); LP-M (men with PSA 2.5–4.0 ng/mL who underwent MRI-TBx); HP-T (men with PSA 4.0–10.0 ng/mL who underwent TRUS-Bx); HP-M (men with PSA 4.0–10.0 ng/mL who underwent MRI-TBx).

Of 5502 men who met the inclusion criteria, 2018 (36.7%) were in the LP-T, 186 (3.4%) were in the LP-M, 2953 (53.7%) were in the HP-T, and 345 (6.3%) were in the HP-M group (Fig. 1). The clinical characteristics of the participants are summarized in Table 1. In low PSA groups (2.5–4.0 ng/mL), there were no significant differences in age and PSA level between the biopsy modalities (LP-T vs. LP-M; *P* = 0.100 and *P* = 0.207, respectively). Similarly, in the high PSA groups (4.0–10.0 ng/mL), age and PSA level were comparable between biopsy modalities (HP-T vs. HP-M; *P* = 0.689 and *P* = 0.907, respectively). Men in the LP-T group were significantly younger than those in the HP-T group (*P* < 0.001). In contrast, the ages of men in the LP-M and HP-M groups were not different (*P* = 0.152). Prostate volume and PSA density showed significant differences in all the comparisons (all *P* < 0.001). There was no difference in the distribution of PI-RADSv2 scores between LP-M and HP-M groups (*P* = 0.105).

**Table 1:** Clinical characteristic of men who underwent prostate biopsy.

Variables	Low PSA groups			High PSA groups			LP-T versus HP-T	LP-M versus HP-M
	LP-T	LP-M	*P* value	HP-T	HP-M	*P* value	*P* value	*P* value
No. of patients, *n* (%)	2018 (36.7)	186 (3.4)		2953 (53.7)	345 (6.3)			
**Age, years**			0.100			0.689	< 0.001	0.152
Mean ± SD	61.9 ± 8.5	62.9 ± 8.4		63.9 ± 9.3	64.1 ± 8.6			
Median (range)	62.0 (33.0–87.0)	63.0 (37.0–81.0)		64.8 (31.0–89.0)	64.4 (31.0–86.0)			
**PSA, ng/mL**			0.207			0.907	< 0.001	< 0.001
Mean ± SD	3.3 ± 0.4	3.3 ± 0.4		6.0 ± 1.6	6.0 ± 1.6			
Median (range)	3.3 (2.5–3.9)	3.4 (2.5–3.9)		5.6 (4.0–9.9)	5.5 (4.0–9.9)			
**Prostate volume, mL**			< 0.001			< 0.001	< 0.001	< 0.001
Mean ± SD	37.3 ± 15.4	32.1 ± 11.5		43.8 ± 21.7	37.2 ± 17.9			
Median (range)	33.9 (12.0–231.0)	29.7 (12.0–83.0)		38.8 (12.0–298.0)	32.6 (5.0–103.0)			
**PSA density, ng/mL**^**2**^			< 0.001			< 0.001	< 0.001	< 0.001
Mean ± SD	0.10 ± 0.04	0.12 ± 0.04		0.17 ± 0.09	0.20 ± 0.11			
Median (range)	0.09 (0.01–0.30)	0.11 (0.03–0.32)		0.15 (0.03–0.58)	0.17 (0.05–0.78)			
**PI-RADSv2 score**^**†**^								0.105
3, *n* (%)		8 (7.3)			18 (9.1)			
4, *n* (%)		72 (65.5)			105 (53.0)			
5, *n* (%)		30 (27.3)			75 (37.9)			

*PSA* Prostate-specific antigen, *TRUS-Bx* Transrectal ultrasound-guided biopsy, *MRI-TBx* magnetic resonance imaging-targeted biopsy, *PI-RADSv2* Prostate imaging reporting and data system version 2, *SD* Standard deviation.

Groups: LP-T (men with PSA 2.5–4.0 ng/mL who underwent TRUS-Bx); LP-M (men with PSA 2.5–4.0 ng/mL who underwent MRI-TBx); HP-T (men with PSA 4.0–10.0 ng/mL who underwent TRUS-Bx); HP-M (men with PSA 4.0–10.0 ng/mL who underwent MRI-TBx).

^†^PI-RADSv2: LP-M (*n* = 110), HP-M (*n* = 198).

**Figure 2:**
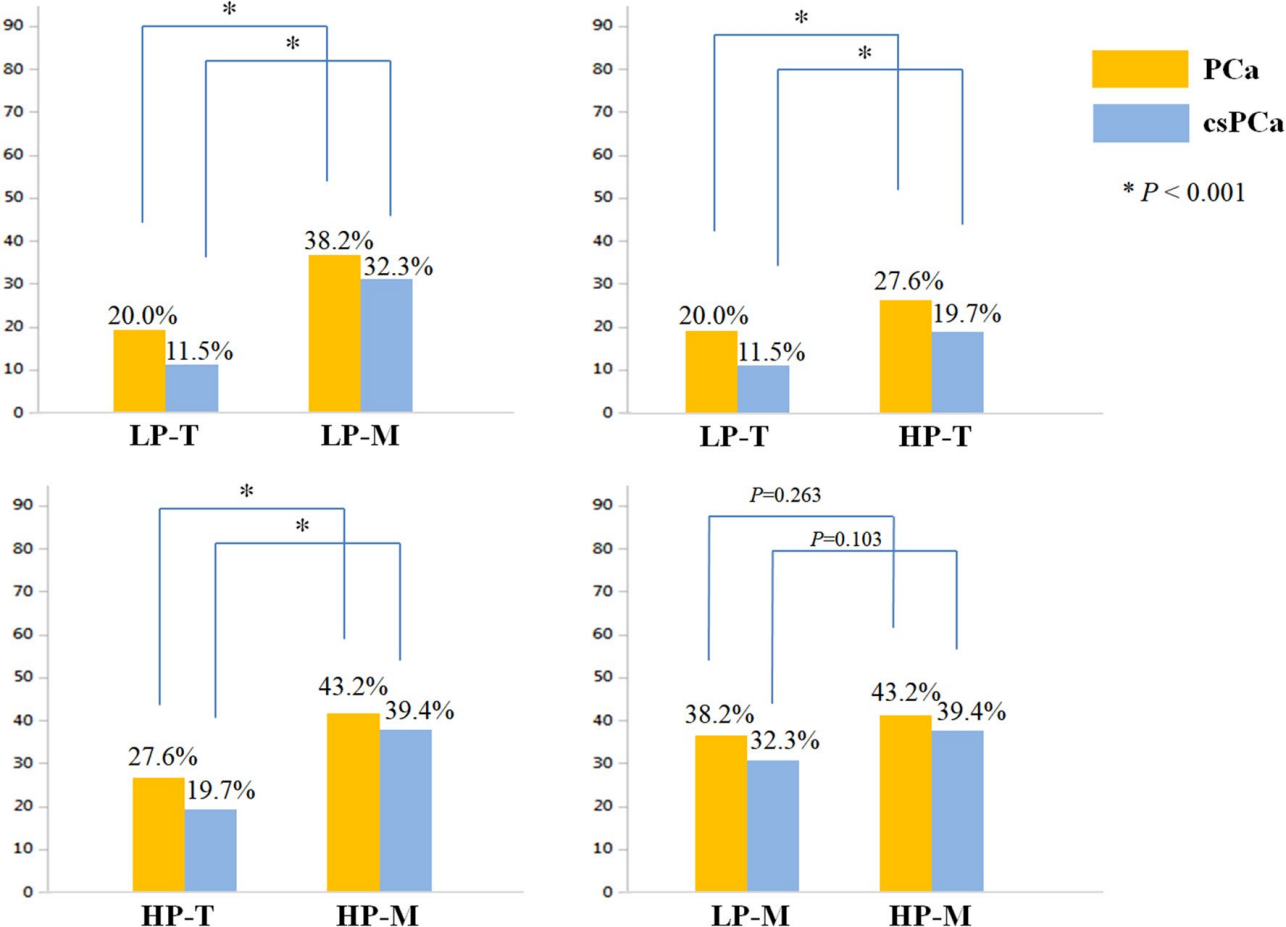
Detection rates of prostate cancer and clinically significant prostate cancer in each group. *TRUS-Bx* Transrectal ultrasound-guided biopsy, *MRI-TBx* Magnetic resonance imaging-targeted biopsy. Groups: LP-T (men with PSA 2.5–4.0 ng/mL who underwent TRUS-Bx); LP-M (men with PSA 2.5–4.0 ng/mL who underwent MRI-TBx); HP-T (men with PSA 4.0–10.0 ng/mL who underwent TRUS-Bx); HP-M (men with PSA 4.0–10.0 ng/mL who underwent MRI-TBx).

The detection rates of PCa and csPCa in each group are presented in Fig. 2. When comparing the detection rates according to the biopsy modality in low PSA groups (LP-T *vs.* LP-M), the detection rates of both PCa (20.0% vs*.* 38.2%; *P* < 0.001) and csPCa (11.5% vs. 32.3%; *P* < 0.001) were significantly higher in the LP-M group than in the LP-T group. Similarly, the detection rates of both PCa (27.6% vs. 43.2%; *P* < 0.001) and csPCa (19.7% vs. 39.4%; *P* < 0.001) were significantly higher in the HP-M group than in the HP-T group. When comparing according to PSA level in men who underwent TRUS-Bx (LP-T vs. HP-T), the detection rates of both PCa (20.0% vs. 27.6%; *P* < 0.001) and csPCa (11.5% vs*.* 19.7%; *P* < 0.001) were significantly lower in the LP-T group than in the HP-T group. In contrast, when comparing according to PSA level in men who underwent MRI-TBx (LP-M vs. HP-M), there were no significant differences in the detection rates of PCa (38.2% vs. 43.2%; *P* = 0.263) and csPCa (32.3% vs. 39.4%; *P* = 0.103) between the groups.

Table 2 shows detailed pathologic biopsy results in men diagnosed with PCa. There were no differences in the total number of biopsy cores in all the comparisons. Except for the comparison between LP-M and HP-M, there were significant differences in the number of positive cores between groups (all *P* < 0.001). There were also significant differences in the maximum percentage of cancer per core between the LP-T and LP-M, HP-T and HP-M, and LP-T and HP-T groups (all *P* < 0.001).

**Table 2:** Pathologic characteristics of men diagnosed with prostate cancer.

Variables	Low PSA groups			High PSA groups			LP-T versus HP-T	LP-M versus HP-M
	LP-T	LP-M	*P* value	HP-T	HP-M	*P* value	*P* value	*P* value
No. of PCa patients, *n* (%)	404 (28.1)	71 (4.9)		814 (56.6)	149 (10.4)			
No. of csPCa Patients, n (%)	232 (23.0)	60 (5.9)		581 (57.6)	136 (13.5)			
**Total number of cores**			0.050			0.040	0.143	0.367
Mean ± SD	11.7 ± 1.2	10.9 ± 3.3		11.8 ± 1.3	11.3 ± 2.8			
Median (range)	12.0 (4.0–14.0)	12.0 (4.0–16.0)		12.0 (4.0–24.0)	12.0 (4.0–16.0)			
**No. of positive cores**			< 0.001			< 0.001	< 0.001	0.101
Mean ± SD	2.7 ± 2.0	4.6 ± 2.4		3.7 ± 2.8	5.2 ± 2.5			
Median (range)	2.0 (1.0–11.0)	5.0 (1.0–13.0)		3.0 (1.0–13.0)	5.0 (1.0–13.0)			
**Maximum % cancer/core**			< 0.001			< 0.001	< 0.001	0.076
Mean ± SD	30.4 ± 25.3	53.0 ± 30.5		42.3 ± 29.8	60.4 ± 25.3			
Median (range)	25.0 (1.0–100.0)	60.0 (5.0–95.0)		40.0 (1.0–100.0)	60.0 (5.0–100.0)			
**Biopsy Gleason score,** ***n* (%)**								
6	238 (58.9)	26 (36.6)		354 (43.5)	44 (29.5)			
7 (3 + 4)	84 (20.8)	29 (40.8)		195 (24.0)	46 (30.9)			
7 (4 + 3)	51 (12.6)	8 (11.3)		102 (12.5)	28 (18.8)			
8	22 (5.4)	8 (11.3)		112 (13.8)	21 (14.1)			
9–10	9 (2.2)	0 (0)		51 (6.3)	10 (6.7)			

*PCa* Prostate cancer, *PSA* Prostate-specific antigen, *TRUS-Bx* Transrectal ultrasound-guided biopsy, *MRI-TBx* Magnetic resonance imaging-targeted biopsy, *SD* Standard deviation.

Groups: LP-T (men with PSA 2.5–4.0 ng/mL who underwent TRUS-Bx); LP-M (men with PSA 2.5–4.0 ng/mL who underwent MRI-TBx); HP-T (men with PSA 4.0–10.0 ng/mL who underwent TRUS-Bx); HP-M (men with PSA 4.0–10.0 ng/mL who underwent MRI-TBx).

The results of the multivariate regression analysis are summarized in Table 3. Age, PSA, prostate volume, and biopsy modality were significantly correlated with the detection of both PCa and csPCa. In men with low PSA levels, it was confirmed that the performing MRI-TBx was significantly correlated with the detection of both PCa [odds ratio (OR) 1.895; 95% confidence interval (CI) 1.355‒2.651; *P* < 0.001] and csPCa (OR 2.872; 95% CI 1.996‒4.132; *P* < 0.001).

**Table 3:** Multivariate logistic regression analyses to determine the predictive factors for the detection of prostate cancer and clinically significant prostate cancer in men with low PSA levels (2.5–4.0 ng/mL).

Variables	Prostate cancer		Clinically significant prostate cancer	
	OR (95% CI)	*P* value	OR (95% CI)	*P* value
Age	1.072 (1.058–1.087)	< 0.001	1.085 (1.067–1.103)	< 0.001
PSA	1.536 (1.178–2.002)	0.002	1.793 (1.288–2.495)	0.001
Prostate volume	0.938 (0.927–0.948)	< 0.001	0.917 (0.903–0.931)	< 0.001
MRI-TBx versus TRUS-Bx	1.895 (1.355–2.651)	< 0.001	2.872 (1.996–4.132)	< 0.001

*PSA* Prostate-specific antigen, *OR* Odds ratio, *CI* Confidence interval, *TRUS-Bx* Transrectal ultrasound-guided biopsy, *MRI-TBx* Magnetic resonance imaging-targeted biopsy.

## Discussion

In the present study, we aimed to investigate the usefulness of MRI-TBx for detecting PCa and csPCa in men with low PSA levels. The detection rates of PCa and csPCa in men with low PSA levels (2.5–4.0 ng/mL) using MRI-TBx were 38.2% and 32.3%, respectively. We found no significant difference in the PCa and csPCa detection rates between the low and high PSA groups when MRI-TBx was performed. Our study indicated that using MRI-TBx rather than TRUS-Bx in men with low PSA levels could improve the detection rates of PCa and csPCa as much as those in men with higher PSA levels. To the best of our knowledge, this study was the largest to demonstrate the value of MRI-TBx for detecting PCa and csPCa in men with low PSA levels (2.5–4.0 ng/mL).

Previous studies have evaluated the detection rate of PCa in men with low PSA levels using TRUS-Bx. Studies from a Western population reported that systematic TRUS-Bx in men with PSA levels between 2.5 and 4.0 ng/mL showed PCa detection rates of 24.5%–27.0%^15,16^. A study of a Japanese population reported a 23.6% detection rate of PCa using systematic TRUS-Bx in men with PSA levels between 2.0 and 4.0 ng/mL^17^. In our study, the detection rates of PCa and csPCa in LP-T group were 20.0% and 11.5%, respectively. Noticeable, those detection rates were significantly lower than those of LP-M groups (all *P* < 0.001).

There is no clear consensus on the diagnostic strategy for PCa in men with low PSA levels. Recent studies (i.e., PRECISION, 4 M, and MRI-FIRST) provided substantial evidence that MRI-TBx is more precise with respect to detecting clinically significant cancer than 10–12 core TRUS-Bx (Table 4)^11–13^. Regarding the MRI-TBx technique, the PRECISION trial conducted targeted biopsy of positive lesions on mpMRI using real-time ultrasonographic guidance without systematic biopsy. The 4 M trial conducted in-bore MR-guided biopsy with 12-core systematic biopsy, and the MRI-FIRST trial conducted both targeted biopsy of positive lesions on mpMRI and 12-core systematic biopsy. Although the MRI-TBx techniques of the studies differed, all three studies consistently showed that the detection rates of GS 3 + 4 or greater PCa using MRI-TBx were higher than those found using standard TRUS-Bx: 38% vs. 26% in the PRECISION trial, 25% vs. 23% in the 4 M trial, and 32% vs. 30% in the MRI-FIRST trial, respectively. However, it should be noted that these three studies investigated populations with a median PSA level of 6.5 or 6.75 ng/mL, which is more characteristic of the men with high PSA levels (4.0–10.0 ng/mL; median: 5.6 ng/mL) than those of the men with low PSA levels (2.5–4.0 ng/mL; median: 3.3 ng/mL) in our study.

**Table 4:** Data from three major manuscripts of MRI-TBx.

	Population	Median PSA	Definition of csPCa	Reference standard	MRI-TBx technique	Detection rate of csPCa by MRI-TBx	Detection rate of csPCa by TRUS-Bx
PRECISION^10^	252 in MRI-TBx 248 in Standard biopsy	6.75 ng/mL in MRI-TBx 6.50 ng/mL in Standard biopsy	GS ≥ 3 + 4	10–12 core TRUS-Bx	Cognitive or fusion targeted biopsy of mpMRI lesion without systematic biopsy	38% (95/252)	26% (64/248)
4M^11^	626 (cohort)	6.4 ng/mL	GS ≥ 3 + 4	10–12 core TRUS-Bx	In-bore MR-guided biopsy with 12-core systematic biopsy	25% (159/626)	23% (146/626)
MRI-FIRST^12^	251 (cohort)	6.5 ng/mL	GS ≥ 3 + 4	10–12 core TRUS-Bx	Cognitive or fusion targeted biopsy of mpMRI lesions with 12-core systematic biopsy	32% (81/251)	30% (75/251)

*csPCa* Clinically significant prostate cancer, *PSA* Prostate-specific antigen, *TRUS-Bx* Transrectal ultrasound-guided biopsy, *MRI-TBx* Magnetic resonance imaging-targeted biopsy.

In terms of the biopsy procedures of MRI-TBx, those used in the present study are most similar to those of the MRI-FIRST trial. We conducted targeted biopsy of positive lesions on mpMRI with 2 or 3 biopsy cores (median, 2 cores; range, 1–4 cores per patient), and an additional 6–10 cores of systematic biopsies were obtained under TRUS guidance. The detection rate of GS 3 + 4 or greater cancer in the HP-M group in our study was 29.6% (102/345), and this was lower than the detection rate in the MRI-FIRST trial (29.6% in HP-M group vs. 37.5% in MRI-FIRST trial). A possible explanation for the lower detection rate in our study may be the differences in disease prevalence within the race of the study population. In addition, fewer total biopsy cores could affect the lower detection rate of GS 3 + 4 or greater PCa in the HP-M group in our study (mean 11.3 cores per patient in the HP-M group; mean 15.4 cores per patient in MRI-FIRST trial). The point to note in our study is that the detection rates of both PCa and csPCa using MRI-TBx did not differ between LP-M and HP-M groups. In addition, MRI-TBx showed a significant correlation in the detection of both PCa (OR 1.895; 95% CI 1.355‒2.651; *P* < 0.001) and csPCa (OR 2.872; 95% CI 1.996‒4.132; *P* < 0.001) in men with low PSA levels. These results indicate that MRI-TBx could yield improved detection rates of PCa and csPCa compared with TRUS-Bx even in a population with low PSA levels (2.5–4.0 ng/mL).

Of note, many studies have shown that mpMRI cannot be used to replace a prostate biopsy. For instance, PI-RADSv2 scores indicate the risk of significant cancer, and scores lower than 3 do not guarantee the absence of csPCa. Park et al. analyzed 119 men with a mean PSA of 5.4 ng/mL and a PI-RADSv2 score ≤ 3 and reported that the detection rate of PCa with biopsy GS of 7 or greater was 23.5% in this group of patients^18^. A research group of radiologists analyzed 100 patients who underwent mpMRI and subsequent radical prostatectomy^19^. They showed that 16% of lesions were missed on mpMRI, and 58% of the missed cancers were either PIRADSv2 score 1 or 2 lesions. In addition, a recent study in the United Kingdom analyzed men enrolled in the PROMIS study to investigate the characteristics of PCa that were overlooked by mpMRI^20^. This study reports that the proportions of clinically important cancers overlooked by mpMRI are 7% and 13%, respectively, according to their definition, and that those cancers had a relatively lower grade and smaller size. As csPCa in men with low PSA levels, which is not visible on mpMRI, has not been fully elucidated, further studies on the characteristics of patients with these cancers and methods of detecting these cancers are needed.

And lastly in order to confirm the recent trend, we selected only cases after 2016 and analyzed them again. The detection rates according to the biopsy modality in low PSA groups (LP-T vs. LP-M), the detection rates of both PCa (20.30% vs. 39.90%; *P* < 0.001) and csPCa (12.30% vs. 34.00%; *P* < 0.001) were significantly higher in the LP-M group than in the LP-T group. In other words, we could confirm LP-M showed superior result from our study cohort after 2016 in cancer detection rate and clinically significant cancer detection rate.

This study has several limitations. First, its retrospective nature and single-institution design may have led to selection bias. Specifically, although our institution has recommended prostate biopsy for all men with PSA levels 2.5 ng/mL or higher since 2008, men in the low PSA groups may be less likely to undergo biopsy than men in the high PSA groups^14^. Beside, PSA level between 2 and 10 ng/ml is considered as grey zone, recently additional examination such as PHI, MRI could be applied. These tests are not routinely performed, they can certainly be helpful, and there are cases where these tests were performed in this study as well. However, as a retrospective study, selection bias is inevitably involved. Second, the interpretation of mpMRI using PI-RADSv2 was introduced in the middle of the study period, so it could be investigated in only a small number of patients. However, this limitation could be overcome by the fact that all MRI-TBx procedures were performed by two radiologists with more than 10 years of experience. Third, the present study focused primarily on pathological findings from needle biopsies; we did not assess pathological findings from prostatectomy specimens or long-term outcomes such as biochemical recurrence after radical treatment.

In conclusion, performing prebiopsy mpMRI and MRI-TBx in men with low PSA levels (2.5–4.0 ng/mL) could detect patients harboring csPCa as much as that in men with higher PSA levels (4.0–10.0 ng/mL). Therefore, it may be necessary to recommend MRI-TBx in men with low PSA levels. Further studies on the long-term outcome of the diagnosis of csPCa in patients with low PSA levels are required.

## Materials and methods

### Study population

This study was approved by the Institutional Review Board of Samsung Medical Center (IRB No. SMC 2019-06-038) and conducted in accordance with the Declaration of Helsinki. Informed consent was waived by the board because of the study’s retrospective design. We reviewed the medical records of 5,932 men with PSA levels of 2.5–10.0 ng/mL who underwent prostate needle biopsy at our institution between January 2008 and December 2018. Men with confounding factors, including a history of previous prostate biopsy (*n* = 172), history of prostate surgery (e.g., transurethral resection of the prostate) (*n* = 98), and use of 5-alpha-reductase inhibitors before biopsy (*n* = 160) were excluded. Ultimately, 5502 patients who underwent prostate biopsy were analyzed in this study (Fig. 1). The study cohort was categorized into four groups according to prebiopsy PSA level and biopsy modality: LP-T [men with low PSA (2.5–4.0 ng/mL) who underwent TRUS-Bx], LP-M [men with low PSA (2.5–4.0 ng/mL) who underwent MRI-TBx], HP-T [men with high PSA (4.0–10.0 ng/mL) who underwent TRUS-Bx], and HP-M [men with high PSA (4.0–10.0 ng/mL) who underwent MRI-TBx].

### Clinicopathological parameters

Clinical variables included age at biopsy, prebiopsy serum PSA level, prostate volume as measured using TRUS, biopsy modality (i.e., TRUS-Bx or MRI-TBx), and the pathologic results from prostate biopsy including the total number of biopsy cores, number of positive cores, maximum percentage of cancer per core, and biopsy Gleason score (GS). Serum PSA levels were measured by immunoassay (ADVIA Centaur PSA; Siemens, Munich, Germany).

Prostate mpMRI was performed within two months prior to biopsy using a 3-Tesla MRI scanner (Intera Achieva TX; Philips Healthcare, Best, The Netherlands) with a phase-array body coil. MRI protocols included T1-, T2-, and diffusion-weighted imaging as well as dynamic contrast-enhanced imaging after intravenous injection of gadolinium diethylenetriamine penta-acetic acid (Gadovist; Schering, Berlin, Germany). The MR images were interpreted by two radiologists specializing in urologic radiology. After the adoption of the Prostate Imaging Reporting and Data System version 2 (PI-RADSv2) scoring system at our institution in May 2015, MR images were scored using a 5-point scale for each patient. PI-RADSv2 assessment categories were defined as a score of 1 to 5, with a higher score indicating a greater likelihood of csPCa^18,21^.

### Biopsy procedures

All patients received antibiotic medication prior to the biopsy procedure. TRUS-Bx was systematically performed on 12 cores, including both the standard and lateral sextant schemes, using an 18-gauge biopsy needle mounted on an automatic biopsy gun in a biplanar ultrasound probe (BK Medical, Herlev, Denmark). This procedure was performed by several urologists with different levels of clinical experience.

MRI-TBx biopsy was performed in a cognitive manner using a high-resolution transducer (C8-4v, Philips Health Care, Bothell, WA, USA) in patients whose mpMRI result was found to be suggestive of PCa. After PI-RADSv2 became available, MRI-TBx was performed for lesions with a PI-RADSv2 score of 3 or higher. Each lesion in prebiopsy mpMRI was cognitively targeted on TRUS, and 2 to 3 biopsy cores (median, 2 cores; range, 1–4 cores per patient), and an additional 6–10 cores of systematic biopsies were obtained. In case, only the index lesion target biopsy is performed and additional systematic biopsy is followed, include PIRADS v2 score 3 ~ 5 lesions to target biopsy and except these lesions, the systematic biopsy is performed. This procedure was performed by two radiologists with more than 10 years of experience in the field of urology radiology, and the biopsy was performed by the radiologist who interpreted the patient's MR images.

### Definitions of csPCa

A diagnosis of biopsy-detected csPCa was defined as follows: (1) GS of 6 (3 + 3) with three or more positive cores, (2) > 50% cancer involvement of GS 6 (3 + 3) cancer, or (3) GS of 7 (3 + 4) or higher^22^.

### Statistical analyses

Quantitative variables are presented as mean (standard deviation, SD) and median (range), and qualitative variables are presented as absolute values (percentage). The groups were compared using chi-square test for categorical variables and Student’s *t*-test for continuous variables. A Bonferroni correction was used to adjust for multiple comparisons of the variables, with statistical significance indicated by *P* < 0.0125. Multivariate logistic regression analyses were performed to identify predictors of the detection of PCa and csPCa in men with low PSA levels, and *P* < 0.05 was considered statistically significant. Statistical analyses were conducted using Statistical Package for the Social Sciences (SPSS) version 21.0 (IBM Corp., Armonk, NY, USA).

## Abbreviations

CI: Confidence interval
csPCa: Clinically significant prostate cancer
GS: Gleason score
MRI-TBx: Magnetic resonance imaging-targeted biopsy
mpMRI: Multiparametric magnetic resonance imaging
OR: Odds ratio
PCa: Prostate cancer
PI-RADSv2: Prostate imaging reporting and data system version 2
PSA: Prostate-specific antigen
TRUS-Bx: Transrectal ultrasound-guided biopsy
